# Extending the mind: a review of ethnographies of neuroscience practice

**DOI:** 10.3389/fnhum.2014.00359

**Published:** 2014-06-06

**Authors:** Tara Mahfoud

**Affiliations:** Department of Social Science, Health and Medicine, King’s College LondonLondon, UK

**Keywords:** ethnography, neuroscience, science and technology studies, human brain project, BRAIN

## Abstract

This paper reviews ethnographies of neuroscience laboratories in the United States and Europe, organizing them into three main sections: (1) descriptions of the capabilities and limitations of technologies used in neuroimaging laboratories to map “activity” or “function” onto structural models of the brain; (2) discussions of the “distributed” or “extended” mind in neuroscience practice; and (3) the implications of neuroscience research and the power of brain images outside the laboratory. I will try to show the importance of ethnographic work in such settings, and place this body of ethnographic work within its historical framework—such ethnographies largely emerged within the Decade of the Brain, as announced by former President of the United States George H. W. Bush in 1990. The main argument is that neuroscience research and the context within which it is taking place has changed since the 1990’s—specifically with the launch of “big science” projects such as the Human Brain Project (HBP) in the European Union and the BRAIN initiative in the United States. There is an opportunity for more research into the institutional and politico-economic context within which neuroscience research is taking place, and for continued engagement between the social and biological sciences.

## Introduction

Thirteen years after the end of the Decade of the Brain (DoB), another decade of studying the brain has been formally launched—the Human Brain Project (HBP) in the European Union, and the Brain Research through Advancing Innovative Neurotechnologies (BRAIN) initiative in the United States. These initiatives promise to develop models and simulations of the brain (animal and/or human) in order to better understand and eventually cure illnesses and diseases such as schizophrenia, epilepsy, and Alzheimer’s. In many ways, the DoB had an impact over how neuroscience research was to develop, and over understandings of the role of the brain in the human body—such as brain plasticity, the ability to explore the relationship between brain chemistry, structure, and function through imaging and other technologies, the influence of genetics on understandings of neurological disorders, how drugs can alter the brain, and different understanding of how visual signals are transmitted and processed.^1^ It is likely that the HBP and BRAIN initiatives will also have an impact on how neuroscience is to develop in the coming decade and what kinds of knowledges become favored in this “new” landscape.

The DoB also spurred on a number of ethnographies of neuroscience laboratories in the United States and Europe—much of which this paper aims to review. While there have also been several historical studies of the neurosciences, as well as reviews of Science and Technology Studies (STS) in general, this review will specifically focus on ethnographic accounts of neuroscience practice. But why an ethnography of neuroscience practice in the first place? Included throughout the paper will be the justifications given by the authors for why ethnographic research is well suited to studying the social context and implications of neuroscience research. The aim here is to show the relevance of ethnographic fieldwork in understanding (1) the context within which neuroscience research takes place and the technologies that make it possible; (2) distributed systems and networks in science and what these can say about the assumptions driving neuroscience research; and (3) the implications of neuroscience research once findings have “left” the laboratory.

These ethnographies have been important to understanding the work of neuroscientists in their laboratories, but the current landscape of neuroscience research is changing. The tools used by researchers, the distribution of data collection and integration (not only across disciplines and groups of researchers within laboratories but also across geographical boundaries), and the contemporary institutional frameworks of research (academic and non-academic) have changed since the DoB. Nowadays there are increased pressures placed on researchers in the natural, social and human sciences on producing knowledge with impact or translational value. And with neuroscience becoming “big science” with the announcement of HBP and BRAIN, more work can be done to study the processes through which knowledge is transformed as it enters, exits and re-enters the laboratory and how these processes shape and are shaped by institutional constraints as well as the broader political economy within which this research is taking place. Ethnographic studies of neuroscience knowledge can potentially offer insight into the relationship between the everyday of scientific practice and reasoning on the one hand and the political and moral economy of science on the other, as well as encouraging conversation between the social and biological sciences, as this special issue aims to do.

It is perhaps notable that some of the pioneers of Science and Technology Studies carried out their fieldwork in neuroscience laboratories, even if they may not have explicitly been presented as such. Bruno Latour and Steven Woolgar’s “Laboratory Life” (1987[1979]) was based on fieldwork in the 1970’s at the Salk Institute, exploring Roger Guillemin’s work on neuroendocrinology. While Latour and Woolgar were not specifically interested in the brain itself in these studies, their attention to objects, artifacts, and the embodiment of scientific practice would have an influence over approaches in the social sciences and in the development of Science and Technology Studies. Lynch (1985) also underwent fieldwork in a “neurosciences laboratory”—a neuroanatomy laboratory—between 1975–1977, and began publishing his work in the mid 1980’s. Lynch (1985) looked at the use of images, diagrams, graphs and other “displays” and how these in turn come to shape the object of research in specific ways, including an analysis of “shoptalk” or how scientists talk about their research. These approaches, and their emphasis on how technologies place limits around how objects come to be understood in scientific practice, no doubt paved the way for a focus on the role of objects in the creation, mediation, and communication of scientific knowledge. The next section will look at how more recent ethnographies and studies of neuroscience laboratories have discussed the technologies and objects used and how these come to shape knowledge in the laboratories.

## Mapping mind onto brain: scanning, selecting, collecting, distributing

A couple of years after the seminal contributions of Latour, Woolgar and Lynch to the field of STS, the DoB was announced by then-President George H. W. Bush, from 1990–2000 (Bush, 1990). The first ethnography to take place since the announcement was Joseph Dumit (2004) fieldwork for his PhD in the History of Consciousness at the University of California, Santa Cruz that he completed in 1995. As Dumit says, in the conclusion of his book Picturing Personhood, “I would like to claim, or propose, that with brain function imaging, we, in the United States, may have entered a space of active negotiation of the basic terms of our categories of the person… The use of these images in thinking about ourselves is in its infancy. We are at stake in this work. How can we not afford to risk jumping in and studying it?” (Dumit, 2004, p. 185). It is, for Dumit, a concern over the implications of the DoB and how categories like “person”, “normal”, “mental health” and others could and would be redefined that spurred on ethnographic work in laboratories. Although the neurosciences were not new at the time, announcing neuroscience as a national project was expected to have an effect over how neuroscience was to develop, how these developments would change how people would come to think of and govern themselves, and how social scientists in turn would come to study and interpret these developments (Martin, 2000; Rose and Abi-Rached, 2013).

Rather than taking inspiration from the work of Latour, Woolgar and Lynch, Dumit (2004, p. 11) drew on Appadurai’s research into the “social life of things” (Appadurai, 1986)—“To trace the various ways in which experiments were designed with assumed categories of people, how they were carried out and interpreted, published in technical and popular literature, and read and incorporated into further experiments, patients’ lives, and everyday notions of personhood”. Dumit’s work provides a comprehensive account and understanding of how Positron Emission Tomography (PET) images come to be through an oral history with some of the technology’s founders, as well as observations of scientists’ work in laboratories and conferences.^2^ It is a great opportunity to hear how and why these technologies were developed in the way that they did, especially now, around 40 years after the development of PET technology. Dumit shows how the selection of research subjects for an experiment was such an integral part of neuroimaging processes in PET at the time—so much so that scientists could claim first authorship on a paper if they found the appropriate subject for a specific experiment. However, Dumit (2004, pp. 60–61) reminds the readers, it is never clear “to what extent an individual is representative of a group”, especially so when the experiments involved small testing groups due to the costs incurred in running them. Consequentially, definitions of the normal, abnormal and pathological are contested due to these technologies, because it is most often the extremes (normal or pathological) of each category that are usually sought after for brain imaging experiments (Dumit, 2004; Abi-Rached and Rose, 2010).

Dumit also paid specific attention to how the shape of the scanner itself to a large extent also defines the types of data collected—each scanner is designed to pick up on different types of information.^3^ Dumit showed how the images obtained are limited in form and content, on the types of technologies used, the research subjects selected, and contingent on historically and culturally situated activity. However, he reminds us through interviews with the PET founders that technologies are used for a specific purpose, to try to answer very specific questions and do not claim to “tell all”. Dumit (2004) claims to show how categories of personhood are “incorporated into further experiments, patients’ lives, and everyday notions of personhood” (Dumit, 2004, p. 11). We are taken from the production of the images to the images’ life outside of the laboratory but are not shown how these “worlds” intersect and exchange, how the images transform. Do the traveling images and studies of brain feed back into how researchers do their work and conceptualize their object of research, and if so how? Morana Alac’s fieldwork from 2002, and Tobias Rees’s fieldwork in and around 2005—both discussed in later sections—would address some of these questions by paying more attention to the use of the objects in the laboratory alongside an investigation of how scientists talk about their usefulness, as well as to how scientists come to speak of themselves as neurological beings (respectively).

Following Dumit’s precedent, Beaulieu’s (2004) fieldwork in neuroscience laboratories, completed as part of a PhD in Science and Technology Studies at the University of Amsterdam in 2000, has looked at the development of “brain mapping” in the 1970’s and 80’s and its consolidation in the 1990’s taking into account the digitization of research and databases and how that has an effect on how knowledge in the neurosciences has been shaped (Beaulieu, 2004). Beaulieu (2001, p. 1) also highlights the interest in localization in neuroscience saying, “in contrast to a focus on processes of mind in time, brain mapping redirected attention to patterns of activity located in the space of the brain”. One of Beaulieu’s arguments is that the DoB had the effect of placing emphasis on building standard models, or atlases of the brain (such as the Talairach coordinate system) that could be used in research—not too dissimilar from the current aims of the HBP although perhaps more modest in ambition. In the 1990’s, there was a belief that developments in information technology could help to build better maps of the human brain because more information could be stored with the digitization of databases (Beaulieu, 2001). In the ongoing HBP, it is developments in computing power (supercomputers) that are envisioned to push neuroscience forward as more information can be integrated, or brought together. In both cases, one of the main concerns is over mapping the “mind” (a category Beaulieu, 2001 argues that has been revived as an object of study since the development of imaging technologies) onto the human brain. As Beaulieu (2001, pp. 2–3) Beaulieu explains, the “biologization of mind in brain mapping takes the social or the environmental rather seriously. It renders these as features of a map”. And as she explains elsewhere, areas of study delegated to the social sciences are now being explored in the neurosciences (Beaulieu, 2003). Beaulieu, like Dumit, is concerned over the status and value of the images produced in the laboratories and how important “processes of technological development and institutional embedding [are] for the constitution of these objects” (Beaulieu, 2000, p. 5). Again, it is a concern over the implications of these images and what they hold—an epistemological concern with the “objectivity” of neuroscience knowledge and an ethical concern with regards to its possible and recorded implications.

In a wry twist on mapping function onto spatial coordinates, and inspired by Pierre Bourdieu’s work on how the division of space can reflect and shape a community’s social organization, Roepstroff (2002) maps the functions of neuroscience work in the laboratory onto the different floors of a newly built building in a Georgian neighborhood in central London that they occupy. He points to how the placement of the laboratory in the basement mirrors the placement of the kitchen in the basement in traditional Georgian houses—this is where things are made (Roepstroff, 2002). Roepstorff mentions that this idea was not refuted by the “natives”, and mentions the description by the “native” of how the research subjects and the experimental data travels through the building from floor to floor—subjects entering the building, and data going up to the top of the building. “This ‘logic’ of the place appears, on closer examination, quite magical. Something (experimental subjects) enters the building, ‘they’ go up, and ‘they’ (the data) exit through the top. This suggests that the house is described—and can indeed be conceived of—as a site of transformation where subjects are turned into data” (Roepstroff, 2002, p. 154). Using this analogy, Roepstroff (2002, p. 154) argues that “the house may therefore be described as a black box, where subjects enter at the bottom and objectivity exits at the top”.

In addition, Roepstroff (2002) outlines the important place “St. John’s House” holds in the brain mapping community—one of the most widely used brain mapping software was developed in this building (or rather by people occupying it). But as Roepstorff mentions, “not everybody in the brain mapping field agrees with the particular framework of ‘St. John’s House’ and there are competing analytical tools, particularly in the US and Canada, each surrounded by an esoteric circle of followers. Several researchers located in the exoteric circle relative to ‘St. John’s House’ complain that the framework is being overtly marketed, and informal, gossipy stories circulate about how articles made with alternative analytical frameworks have had problems getting through a peer-review process” (Roepstroff, 2002, p. 159). In this and other work, Roepstorff includes vivid descriptions of the scanning process, how people and data move through the space, and how the data is then analyzed. What his ethnographic work does not manage to address, however, is how the data collection and analysis, which Roepstorff describes as being “black boxed” is actually negotiated between the scientists in the laboratory and the computers they work with. These moments of coordination are explored by Alac and described in a later section.

Roepstorff also draws on Ludwig Fleck’s concept of the “thought style” as “characterized by common features in the problems of interest to a thought collective, by the judgment which the thought collective considers evident, and by the methods which it applies as a means of cognition” (Fleck, 1978, p. 98 cited in Roepstroff, 2002, p. 158). Indeed, usually each laboratory develops its own software, depending on which data they need extracted from the scans. Many different groups of researchers are formed around the same technology, and this is noticeable by researchers because “they have their own source of funding and their own interests” (Dumit, 2004, p. 56). In this sense, “software permits the user to look only at what neuroscientists call “regional interest” rather than allowing him/her to inspect images of the whole brain” (Alač, 2006, pp. 152–153). Collection of data is always attributed to the scanner, rather than to people, and is regarded as a “black-boxed” procedure. Indeed, even authors on a paper will many times not be able to answer questions about how data was collected, or what scanning software was used, as neuroscience itself is such a distributed science, reliant on so many different fields of expertise with many people contributing to the knowledge needed to produce a scientific paper that can be published in a reputable journal (Dumit, 2004). This is why scientific institutional processes have been so often studied as distributed systems.^4^ It becomes difficult to bound attribution to a single body, especially as the boundaries between mind, body and world become ever harder to place and since collective activity involves so many external artifacts. This is the spirit behind discussions of collective agencies, and many of the observations made in science and technology studies—that it is not quite possible to “trace” origins for an action back to a single originator if actions are always entangled. Indeed, the HBP sees this distribution of knowledge as a hindrance to the development of knowledge within the neurosciences, saying:

We find that the major obstacle that hinders our understanding the brain is the fragmentation of brain research and the data it produces. Modern neuroscience has been enormously productive but unsystematic. The data it produces describes different levels of biological organization, in different areas of the brain in different species, at different stages of development. Today we urgently need to integrate this data—to show how the parts fit together in a single multi-level system (Human Brain Project: A Report to the European Commission, 2012, p. 3).

The distribution of knowledge and cognition is a strategy within scientific work in order to collect the necessary amount and kinds of data. But this distribution also has an unintended consequence—knowledge becomes distributed across geography, and the effects of unstandardized technologies make it difficult to “fit together” all the work being done in the expanding field of neuroscience. The next section will explore how this distribution of action within laboratories has been understood in ethnographic studies of neuroscience laboratories.

## Learning to see: vision and the extended/distributed mind

It is thus perfectly possible that certain psychological phenomena cannot be investigated physiologically, because physiologically nothing corresponds to them (Ludwig Wittgenstein 1967, cited in Martin, 2010, p. 366).

The argument made by the authors that appear later in this section is that while brain imagers work with certain assumptions, namely that the “mind” and “cognition” can be mapped onto a simulated model of the brain and matched to specific locations or coordinates, the neuroscientists’ work in the laboratory suggests that the researchers’ environment contributes to this “cognitive” process—even if not explicitly accounted for in their descriptions of the research process. The authors pay attention to the distribution of work across people and artifacts, and to the interaction between bodies and their external environment. In doing so, these studies show how ethnographic work in laboratories can unsettle some of the assumptions driving neuroscience research, in particular the search for the mind only within the boundaries of the human brain.

Based on 9 months of fieldwork at three neuroscience laboratories at the University of California in San Diego and the Salk Institute studying the neural correlates of vision, Morana Alac pays attention to the objects in circulation during a “cognitive” task, as well as the types of sensory-motor information managed by the individuals involved (vision, gesture, touch, posture). Hutchins (1995, p. xiv) (Alac’s PhD supervisor) ideas on “distributed cognition” were born out of long-term fieldwork aboard U.S. Navy ships, where his aim was to understand “how people go about knowing what they know and the contribution of the environments in which the knowing is accomplished”. The title of the book, “Cognition in the Wild’ refers to “the distinction between the laboratory, where cognition is studied in captivity, and the everyday world, where human cognition adapts to its natural surroundings”. This refers to the problem of laboratory work, and the problem of generalizing findings to human behavior outside of it.

Drawing on Hutchin’s work, Alač (2006, p. 10) says studying cognition in social settings (rather than as a process internal to the individual) offers many advantages; “a study of cognitive processes of a single individual does not always allow one to predict the properties of the system. A distributed cognitive system may have emergent characteristics that are generated by interactions among properties that are not present in any individual element of the system”. The distributed action, along with the visualization of the scanner’s data, allow scientists to make sense of the large amount of data retrieved from the scanners and provides a way for scientists to discuss the abstract data in concrete ways. It is not just that action or cognition is already distributed, but scientists intentionally distribute cognition across multiple platforms (human and non-human) in order to solve the problem of wanting to see inside the human brain. Alac draws on Edwin Hutchin’s theory of “distributed cognition” to explain how Octavia, a scientist in the laboratory, uses a chart that she developed and drew by hand to see and teach other people to see cognitive processes in brain images—specifically the distribution of activity over time, and the motion of activity across the mapped brain. Octavia uses the chart and her hands to gesture and to point at the screen, explaining to the “novice” that “it takes quite a bit of training to start and actually see” (Alač and Hutchins, 2004; Alač, 2006, p. 118).

The meaning of these brain images, although reliant on scientific knowledge (as communicated through the charts prepared by the “expert”), also require technical skill and a social setting for that meaning to be made. This marks a break between the types of knowledge scientists gain in formalized education, and that gained “in practice”, as other anthropologists have observed in medical practice.^5^ We also see how important the representational material (for example, the chart made by the expert) is to the entire process of knowledge production. Making sense of the breadth of data represented in these images requires the mobilization of “cognitive artifacts” as Hutchins (1999) calls them—objects that help people perform cognitive tasks such as understanding. Alac shows how this process is one of “distributed cognition”, whereby the end product, which is an understanding of what the brain images show, is gained through interactions with objects (the computer, the chart) and people. But it is also such an embodied practice, that it requires an “expert” to teach the “novice”, rather than the “novice” being able to learn through representational material alone.

In a related manner, Andreas Roepstorff discusses the concept of “skilled vision”, and how seeing certain things in brain images require skills that are learned in a specific social context. At the laboratory where Roepstorff works, they have invited a “master” neuropsychologist to look at some of their experimental data. To test his knowledge, they give him a couple of images and ask him to figure out what the experiments were with no prior knowledge of the set-up. By looking at the areas of activation as shown in the images, the neuropsychologist is able to tell what the scientists at the laboratory were testing for in their experiments. Roepstorff uses this example to show how the “master” neuropsychologist could not only interpret brain images “on the fly”, but also is able to place them in a narrative and begin to explain the relationship between both images and what the data “means”. While Roepstorff does not look at the artifacts used to aid cognition and their role in distributed cognition as Alac does, he is paying attention to the social context that comes into play when “learning how to see”. Roepstorff concludes that rather than using vision in order to know things, in neuroscience laboratories one needs to know in order to see. The neuropsychologist’s ability to locate significant data on the maps/brain images points to how brain images contain within them a limited number of ways the data can be interpreted, and one’s knowledge of the markers reinforces one’s belonging to a brain imaging community. Seeing, especially in laboratories, is usually thought of as sensory in nature rather than social. And the assumption that seeing is knowing, argues Roepstorff, lies behind how powerful brain images are in their ability to provide a window into the human brain. By arguing that seeing requires knowledge about an issue means that “seeing” requires a social context within which sight can be formed or taught.

“Cognitive processes ain’t (all) in the head!” say Clark and Chalmers (1998) Chalmers in an article titled “The Extended Mind”. Their argument is that in certain situations, “the human organism is linked with an external entity in a two-way interaction, creating a coupled system that…counts equally well as a cognitive process, whether or not it is wholly in the head” (Clark and Chalmers, 1998, p. 9). Research by Nicolas Langlitz into the use and experience of the effects of psychedelics (lysergic acid diethylamide (LSD) and psilocybin) in laboratories in Switzerland, Germany and the United States has shown how important the setting or context is to how an individual reacts to the influence of the drugs ingested. Langlitz mentions how no pharmaceutical company is interested in psychedelics, for example, because there is no way of controlling how a person is to react—it is dependent on so many things within the individual and outside of him/her. Pharmaceutical companies want to find drugs that work the same on everyone, which targets a very specific location in the brain. What Langlitz is saying is that the mind cannot be reduced to the brain alone, given how important surroundings are to individuals’ experiences and cognitive behavior and to how unpredictable the social context will be at a given time, referring to the “extended mind hypothesis” (Langlitz, 2013).

In a similar vein, Simon Cohn (2008, p. 90) argues that, “what finally appears as an area of activation in the final brain scan of a volunteer is actually the combined response of the person in the scanner, the physical provisions of the experiment, and the thinking of the scientist who not only has to prime the volunteer, but who also establishes an essential level of intimacy with him or her in order for the experiment to be conducted in the first place”. Adding to the discussions by Alac, Beaulieu, and Dumit who show how the interactions with technologies and artifacts shape the ways in which interpretations are made in laboratories, Cohn is showing how the interactions between the scientists running the experiments and the volunteers create a certain environment, or “level of intimacy” that shapes the environment within which the experiments are performed. If the “mind” is inseparable from body and world, the human (and non-human) environments within which cognition take place are as essential to understandings of how the body works. Ethnographic research can unsettle findings of neuroscience research because it is able to pay attention to interactions between bodies, artifacts, and their environments. As Alač (2011, p. 162) says, her work in the laboratories “has moved to unpack two theoretical assumptions that have dominated the examined historical period in cognitive neuroscience: the idea that human cognition is internal, and the assumption that embodiment concerns a single person and, in particular, her or his brain processes”. But if cognition and the “mind” are distributed and extended, how far are they extended and how much of a person’s environment can be included? The next section will look at the importance of looking into the institutional framework within which neuroscience research takes place, as part of the environment involved in the thinking of researchers in neuroscience.

## How far out of the lab: the links between research production and the management of research

In the last two chapters of “Picturing Personhood”, Dumit (2004) moves his gaze from what is taking place within the lab to looking for where the brain images “travel” to once they have left the laboratory. These chapters do not rely on ethnographic observation, and instead look to the implications of using brain images in courtrooms and how images are presented to the media using historic case studies and representations in popular media such as film. While providing a useful overview of the controversy of showing medical images in courtrooms, future ethnographic accounts of the presentation and interpretation of these images in court could offer more insight into how the meaning is negotiated within this context between the “experts”, legal staff, and jury—that is if an ethnographer could gain access to such settings. Similar to how Cohn (2010) speaks to the subjects of neuroscience experiments in order to understand their own perceptions and interpretations of their brain scans, interviews and fieldwork in courtroom cases that use brain images as “evidence” can show the disjuncture between “expert” and “popularized” interpretations over the meaning of these images as well as the negotiation over the implications of such medical knowledge and how these will come into play in judging a person’s culpability.

Another important part in how images leave the laboratory is in the publication and peer review process. Alač (2004) describes the process of submitting, reviewing and publishing an article in the journal Science. The authors of the article are researchers in the same laboratories Alac conducted her research in. These researchers have developed their own software, which inflates and flattens cortical maps. While the software is becoming more commonly used in visual cortex mapping, Alac shows how different and novel ways of representing data have to be negotiated with their peers throughout the review process. Alač (2004, pp. 201–202) argues that “the meaning of the text, and especially the results of the study, are in a significant manner produced through the brain images reproduced in the article” in the form of comments and amendments to how the data is presented and in how “each single image acquires its meaning by referring to the other images that are part of the figure”. This is reminiscent of Anne Beaulieu’s (2004) comment that the individual brain image becomes valuable in relation to the other images in the datasets. Alac shows that the experimental methods adopted by the scientists are met with resistance from the reviewers, who are requesting for the data to be visualized in more familiar, conventional ways so that they are more accessible and easily understood. This negotiation, taking place through the images (comments about and amendments to), is not about the esthetics of the image, but about the criteria, or parameters, underlying the ways in which the images have come to be. Paying attention to the negotiation between the reviewers and the researchers shows how important moments of coordination are in understanding how information changes as it exits and re-enters the laboratory.

In a more explicit focus on the political economy of neuroscientific research, Langlitz’s fieldwork in a human lab in Switzerland and an animal lab in California (both working on the effects of psychedelics such as LSD and psilocybin) shows how powerful national policies and legal frameworks can be in deciding what areas are deemed valid for scientific inquiry over others. For example, research with psychedelics on humans has not been possible outside Switzerland because of the drug regulation laws and policies in the US banning the use of illegal drugs in research. Langlitz shows how the hype around neuroscience research following the DoB allowed for a “revival of hallucinogen research” in Europe. This comparative and multi-sited examination of the institutional constraints and the broader political economy in Switzerland, Germany and the United States—national and international drug regulations, pharmaceutical markets, institutional and ethical review boards, and the hospitals housing the laboratories—is especially important within the framework of the newly launched HBP since every country within the EU has different laws and policies regulation research practice and uses of data.

These ethnographies show how the institutional settings of neuroscience, and the complex web of networks and actors this invokes, play into the development of “cognitive artifacts” (the tools favored and used in neuroscience research), in defining researcher interests and priorities, and in defining what is to be considered “noise” versus valid and usable data (what is included in the datasets being developed in laboratories). Indeed, looking for “zones of interest”—what is important to look at—inevitably excludes other “zones”. However, and like any empirical discipline (including ethnography), there is always a need to define the variables and limit the amount of information collected and analyzed—to “reduce noise”. This is not an attack on the validity of neuroscience findings since this is arguably shared across all disciplines, but is rather an opportunity to explore how contemporary concerns become embedded into the accumulation and interpretation of research findings. Concern is expressed over how findings in neuroscience are generalized from the individual (also many times animal) to the collective human (Rose and Abi-Rached, 2013), and this is especially problematic when considering the pressure researchers are under to show the usefulness of their research in order to maintain funding. Although this is not a problem specific to the neurosciences, this limitation has been readily acknowledged by neuroscientists as is indicated in the following quote: “we know we need hype to sell our research; let’s try to keep it out of the results!” (Louis Sokoloff cited in Dumit, 2004, p. 53). These “translational” issues (Rose and Abi-Rached, 2013) of how information is mediated in its traverse from idea to experiment to publication, presentation, etc., can be further studied ethnographically.

For example, Rayna Rapp’s work explores the translational research imperative (from bench to bedside) that has become a condition for funding in the US, and how this affects the hype of medical research, how the media represents the findings of research and how people in turn come to understand and define who they are. Rapp (2011b) follows the “laboratory labors of two scientific groups: neuroscientists who scan children’s brains in search of resting state differences according to diagnosis and psychiatric epidemiologists who look to epigenetics to distinguish differential diagnostic populations” Rapp (2011b, p. 662). Rapp shows how powerful medicalized understandings of learning difficulties are in how parents and their children come to understand differences in development and children’s behavior, and in turn to how researchers understand and develop their own work. Rapp warns against the dangers of hyped research and the hopes it embodies, and reminds readers of the changing trends in research—that these understandings of the mind and how it works shall pass and change, as the institutions that support (and are supported by) research change.

In a similar vein (although not explicitly mentioning issues of translation), Tobias Rees investigates the relationship between life and science in the laboratory of Alain Prochiantz in Paris, France. Rees (2010) in concerned with how plasticity, as a technological and conceptual development in the history of the neurosciences, has become an ethical concern: “human beings cease to be fixed and immutable machines, cease to be already wired information-processing computers of sorts” (Rees, 2010, p. 157). Rees shows how scientists in the laboratory he studies come to define themselves as neurological beings, using the same terms they use to understand their object of research, and how powerful these concepts (such as plasticity) become in how the relationship between life and science become in answering what Rees defines as some of the key questions in ethics: “What shall we do? How shall we live?” (Rees, 2010, p. 159).

Over a decade after the DoB, there is a similar promissory tone to the announcements of the HBP in the EU and the BRAIN project in the US. The brain is presented as the last frontier in human science—the last piece in the puzzle to understand how the human body works together—and justifications for undertaking such expensive and expansive research projects typically refer to the impact of “disorders of the brain”. The Proclamation, made in 1990 by former President George W. Bush states:

Over the years, our understanding of the brain—how it works, what goes wrong when it is injured or diseased—has increased dramatically. However, we still have much more to learn. The need for continued study of the brain is compelling: millions of Americans are affected each year by disorders of the brain ranging from neurogenetic diseases to degenerative disorders such as Alzheimer’s, as well as stroke, schizophrenia, autism, and impairments of speech, language, and hearing.

Thirteen years after, the justifications of the HBP are not so different:

These technologies can enormously accelerate brain research. They can also open the road to treatments that prevent and cure brain disease and to new computing technologies with the potential to revolutionize industry, the economy and society. Medical informatics can mine enormous volumes of clinical data allowing us to understand the basis causes of brain diseases, a pre-condition for early diagnosis, prevention and cure… If European industry is to play a leading role in the world economy of the 2020s and 2030s, it has to take the lead in developing these technologies (Human Brain Project: A Report to the European Commission, 2012, p. 8).

This statement refers not just to the importance of finding better cures for “brain diseases” but also the important economic role that the HBP would play in developing European industry. In response, perhaps, the US launched the BRAIN Initiative and although different in scope and approach, in a section on the National Institutes of Health (NIH) website dedicated to the BRAIN Initiative, there is a link on the left of the screen titled, “Why is this needed?” that states:

With nearly 100 billion neurons and 100 trillion connections, the human brain remains one of the greatest mysteries in science and one of the greatest challenges in medicine. Neurological and psychiatric disorders, such as Alzheimer’s disease, Parkinson’s disease, autism, epilepsy, schizophrenia, depression, and traumatic brain injury, exact a tremendous toll on individuals, families, and society…If we are ever to develop effective ways of helping people suffering from these devastating conditions, researchers will first need a more complete arsenal of tools and information for understanding how the brain functions both in health and disease (National Institutes of Health, 2013).

Again, justifications for the research bring up how developments in modeling the brain can help better understand how the brain works and in turn how this can help understand “neurological and psychiatric disorders”. As Rose (2013) mentions, “the belief in the implications of advances in the life sciences for our everyday lives is exacerbated by the “translational imperative”—the obligation on researchers in biology and biomedicine to promise to funders, to research assessors, to their university press offices and to the media that the results of their work on the fly, the worm, the mouse or the macaque will soon reach the clinic—usually “in 3–5 years” (Rose, 2013, p. 7). This pushes researchers to think not only of the process of producing knowledge, but also of how that knowledge can or will be managed and used once it has been published.

There is an “oscillation between the condition of knowing through investigation (research) and the condition of asking what is to be done with that knowledge (management)” (Strathern, 2006)—such as the links between the process of producing neuroscience knowledge (Roepstorff, 2001; Alač, 2004, 2005, 2006, 2008, 2011; Alač and Hutchins, 2004; Beaulieu, 2004), and how neuroscience data is used in court rooms, for example (Dumit, 2004). At a time when funding is becoming evermore dependent on researchers showing how impactful their research is, an investigation into the institutional mechanisms supporting (and being supported by) scientific research (ethical review processes, national funding bodies, government agencies, universities, researchers, students, etc.) can provide valuable insight into how the boundaries between research and management are made and crossed. As Strathern (2006, p. 194) notes, “the researcher turns into a manager…when boundaries of expertise are crossed and research has to be presented to those who do not share that ‘everything else”’. What are the implications of this crossing of boundaries, from research production to research management? And how do these two processes affect and relate to one another? How do considerations and ideas about how the management of research is to be done come into play during the research process itself, in deciding what words to use in funding proposals, developing the tools and technologies used in research (the “cognitive artifacts”), and in how researchers “think” of their projects, as is encouraged by funding bodies? Further ethnographic work in the spaces branching out of and into the neuroscience laboratory can maybe help to answer some of these questions.

## Conclusion

From time to time, new forms emerge that have something significant about them, something that catalyzes previously present actors, things, institutions into a new mode of existence, a new assemblage, an assemblage that makes things work in a different manner (Paul Rabinow 2000, cited in Rapp, 2011a, p. 663).

It is a unique opportunity to be able to watch how ethnographic work will develop alongside the developments in neuroscience within the frameworks of the HBP and BRAIN. While the focus has been mainly on brain imaging in these ethnographies, neuroscience has been developing other “non-invasive” tools such as Transcranial Magnetic Stimulation (TMS), and has been developing methods in computational neuroscience that are likely to change the objects and artifacts that comprise the “extended mind” of the researchers in laboratories. For example, the Talairach atlas became a convention in neuroscience in the 1990’s—how will the models and simulations developed as part of the HBP change and shape the way neuroscience research is performed? In addition, the majority of these ethnographies have taken place in laboratories in the US and the UK, but these two countries are marginal actors in the HBP—Switzerland, Spain, France, and other countries will be the main sites of laboratory research. There will be an increased need to understand the distribution of action and research across geographical boundaries, as well as disciplinary.

As is rightly noted by Rose (2013, pp. 23–24), there is a need to “move beyond description, commentary and critique, beyond the study of downstream ‘implications’ of biology and biomedicine, to develop an affirmative relation to the new ways of understanding the dynamic relations between the vital and its milieu… one that seeks to identify and work with those arguments that recognize, in whatever small way, the need for a new and non-reductionist biology of human beings and other organisms in their milieu, and which can thus be brought into conversation with the evidence, concepts and forms of analysis developed in the social and human sciences”. There are several initiatives underway that try to bridge the human, social and biological sciences within the context of neuroscience research.

Roepstorff (2012), now working alongside neuroscientists, looks for ways in which the methods of anthropology can be of aid/use to neuroscience but also how knowledge from both disciplines can cross over. One such approach is the work of Robert Turner, the son of anthropologist Victor Turner, who had an important role in developing fMRI technology into what it is today. Holding a postgraduate degree in anthropology from University College London (UCL), Turner has written about brain plasticity. In an article titled, “How Collective Representations Can Change the Structure of the Brain’, Turner and Whitehead (2008, p. 43) “present recent imaging research which…demonstrates that collective representations can have well-defined cortical representations”. But like Rose and Abi-Rached (2013, pp. 141–163), the authors claim that arguments that there is a part of the brain that is responsible for the “social” side of human behavior is rather simplistic and does not even correspond to neuroscientific data. Turner and Whitehead (2008, pp. 47, 51) accept that even the production of neuroscientific knowledge itself takes place within a social context, saying that and referencing Roepstorff, “in order for the subject to feel comfortable enough in the unfamiliar, confined and very noisy space of the scanner bore, they must be put at ease and reassured by the radiographer, using well-rehearsed social skills. This has aspects of an initiation, a rite of passage”. But they do argue that collective representations^6^ like language do have a cortical basis, saying that much research suggests that repeated activity if done over enough of a period of time, will increase the size of the area in the cortex that was associated with the activity from the first scan, showing that “our brains are reorganized by repetitive motor practice”. This is an opportunity to explore the correspondence between repeated embodied motion, as takes place in the laboratories when “experts” teach “novices” how to read the brain images for example, and attempts by neuroscientists to find the neural correlates of specific activities in the brain. Such an approach, however, “mainly concerned with identifying links between structures in minds and structures in the world” (Roepstorff and Frith, 2012, p. 108), runs the risk of essentializing the social and “may be unable to capture humans as persons” which would separate such an approach from anthropological concerns (Roepstorff and Frith, 2012, p. 108).

But Roepstorff (2012, p. 108) says that what is more important than a hybrid discipline joining anthropology and neuroscience, is the “need to develop a metalevel discourse that can grasp what happens when experiments and concepts travel”. This can, no doubt, be interpreted in several ways, but an attempt at understanding the crossing of boundaries in how research findings are made, how they are presented, and how the implications of research define and re-define human ways of thinking of or doing things may contribute to this project. The Critical Neuroscience initiative which aims to increase collaboration between neuroscientists and social scientists on specific issues and study the social context and implications of neuroscience research (as the title of this special issue suggests); Steven Woolgar and Tanja Schneider’s 3 year research project on the development and implications of neuromarketing; Felicity Callard and Daniel Marguiles’s work on the resting brain; the Foresight and Responsible Research Innovation Lab (FRRIL) studying the implications of the HBP headed by Nikolas Rose; amongst others, can offer some insight into “what happens when experiments and concepts travel” (Roepstorff and Frith, 2012, p. 108).

Rose’s call to “work with these researchers…and guard against the rush to demand immediate impacts in social policies and practices” warns against rehashing critiques of the neurosciences without creating a productive communication between the disciplines (Rose, 2013, p. 19). Observing the ways in which concepts and theories are embedded in objects that travel into and out of the laboratory, understanding the institutional context and constraints that allow objects to develop in specific ways, and how these in turn come to be used by scientists in thinking through understandings of their object of study may help to create an awareness and understanding of the links between research practice and the promotion of research that can be useful in other impactful disciplines beyond the neurosciences.

## Conflict of interest statement

The author declares that the research was conducted in the absence of any commercial or financial relationships that could be construed as a potential conflict of interest.

## Footnotes

^1^“A Decade after the Decade of the Brain”, is a series of interviews with seven directors of NIH funded research on brain-related issues about the impact of advances in the neurosciences since the announcement of the Decade of the Brain, published on February 26, 2010 by the Dana Foundation. Available at: http://www.dana.org/Cerebrum/Default.aspx?id=39435

^2^Dumit visited labs at Washington University in St. Louis, Johns Hopkins University, Brook-Haven National Laboratory, Massachusetts Central Hospital, University of California, Los Angeles, and the University of California, Irvine. Dumit also attended the following conferences: Radiological Society of North America, Society for Neuroscience, Society of Nuclear Medicine, and the Organization for Human Brain Mapping.

^3^This concern has been expressed within the framework of the HBP, since the standardization of data from multiple labs and clinics is necessary to fulfill the main aim of the project—the integration of data in order to build models and simulations of animal and human brains.

^4^The work of Knorr-Cetina (1999), Latour and Woolgar (1987[1979]), Lynch (1985), amongst others have all discussed science as a process distributed across people and objects.

^5^For example, Tom Rice (2010, p. 44) has conducted ethnographic fieldwork in St Thomas’s Hospital in London to study how stethoscopic listening is taught as an “apprenticeship in hearing”.

^6^Borrowing Durkheim’s term and defined in the text as “components of human life that have meaningful existence only because we agree that they do—such as customs, money, religion, cosmology, language, games, laws, power structures and artistic genres” (2008, p. 43).
